# Improving Antibody‐Tubulysin Conjugates through Linker Chemistry and Site‐Specific Conjugation

**DOI:** 10.1002/cmdc.202000889

**Published:** 2021-02-12

**Authors:** Joseph Z. Hamilton, Thomas A. Pires, Jamie A. Mitchell, Julia H. Cochran, Kim K. Emmerton, Margo Zaval, Ivan J. Stone, Martha E. Anderson, Steven Jin, Andrew B. Waight, Robert P. Lyon, Peter D. Senter, Scott C. Jeffrey, Patrick J. Burke

**Affiliations:** ^1^ Seagen Inc. 21823 30th Drive SE Bothell, WA 98021 USA; ^2^ Department of Chemistry University of Illinois at Urbana-Champaign 505 South Mathews Ave. Urbana, IL 61801 USA; ^3^ Protein Sciences Discovery Biologics Merck Research Laboratories 213 E Grand Ave. South San Francisco, CA 94080 USA

**Keywords:** Antibodies, cancer, drug delivery, glucuronides, tubulysin

## Abstract

Tubulysins have emerged in recent years as a compelling drug class for delivery to tumor cells via antibodies. The ability of this drug class to exert bystander activity while retaining potency against multidrug‐resistant cell lines differentiates them from other microtubule‐disrupting agents. Tubulysin M, a synthetic analogue, has proven to be active and well tolerated as an antibody‐drug conjugate (ADC) payload, but has the liability of being susceptible to acetate hydrolysis at the C11 position, leading to attenuated potency. In this work, we examine the ability of the drug‐linker and conjugation site to preserve acetate stability. Our findings show that, in contrast to a more conventional protease‐cleavable dipeptide linker, the β‐glucuronidase‐cleavable glucuronide linker protects against acetate hydrolysis and improves ADC activity *in vivo*. In addition, site‐specific conjugation can positively impact both acetate stability and in vivo activity. Together, these findings provide the basis for a highly optimized delivery strategy for tubulysin M.

## Graphical abstract

**Stability and activity**: Antibody‐tubulysin conjugates were evaluated for activity and acetate stability as a function of linker chemistry and conjugation site. Results show the glucuronide linker and S239 engineered cysteine conjugation preserve the acetate in circulation and improve *in vivo* activity relative to a dipeptide linker and endogenous cysteine conjugation.

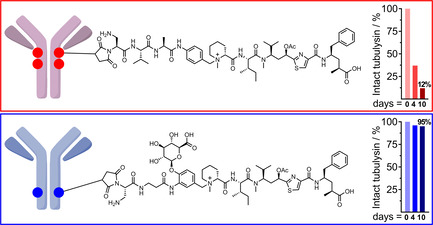

Antibody‐drug conjugates (ADCs) are a therapeutic modality for the treatment of cancer that continue to expand their clinical importance.^[1]^ Recent FDA approvals of two camptothecin‐based ADCs^[2]^ demonstrates the value in expanding the breadth of ADC payloads beyond the clinically successful auristatins,^[3]^ maytansinoids,^[4]^ and calicheamicins.^[5]^ Tubulysins are potent antimitotics that disrupt microtubule dynamics leading to apoptotic cell death.^[6]^ We have shown the synthetically tractable analogue, tubulysin M, is a highly active ADC payload when released in an unmodified state via a quaternary ammonium linkage on the N‐terminal tertiary amine.^[7]^

A potential shortcoming of tubulysin M as a long‐circulating ADC payload is the presence of a hydrolytically labile acetate at the C11 position, the loss of which leads to a significant decrease in biochemical and cytotoxic activity.^[8]^ Conversion of the C11 acetate to a stabilized functional group is a common strategy to circumvent this. For example, replacement with circulation stable moieties like ethers,^[9]^ carbamates^[10]^ and hindered esters^[11]^ may lead to potent tubulysin derivatives. However, these stabilized analogues often underperform as ADC payloads relative to the parent C11 acetate congeners.^[12]^ In this work, we report the use of drug‐linker design and site‐specific antibody conjugation as strategies to stabilize the C11 acetate of tubulysin M while maintaining high levels of ADC activity.

The C11 acetate present on tubulysin M is an important structural feature for maintaining cytotoxic activity. To confirm the impact of the acetate on free drug activity, deacetylated tubulysin M was synthesized (Scheme S4 in the Supporting Information) for *in vitro* comparison. The deacetylated tubulysin M construct showed a >100‐fold loss of cell growth inhibition when compared to tubulysin M against MDR+ renal cell carcinoma cell line 786‐O (Figure 1A). This trend was consistent across a panel of leukemia, lymphoma, and carcinoma cell lines (Table S1).

**Figure 1:**
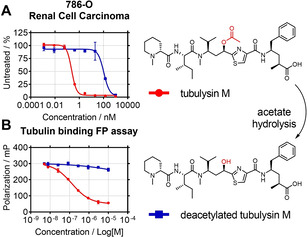
A) Relative free drug cytotoxicity ±SD and B) tubulin binding affinity of intact and deacetylated tubulysin M ±SD.

The impact of the acetate on free drug binding to tubulin was established using a tubulin‐based fluorescence polarization assay in which the drugs were compared in their abilities to displace a fluorescently labeled, high affinity monomethyl auristatin F probe from tubulin. Consistent with the cytotoxicity results, the deacetylated analogue was noncompetitive in the assay indicating a significant decrease in tubulin binding affinity upon the loss of the C11 acetate (Figure 1B).

Quaternary ammonium‐linked tubulysin ADCs were designed to be conjugated via a cleavable ValAlaPAB dipeptide sequence. Beginning with tubulysin M protected via an allyl ester on the C terminus (**3**), synthesis progressed in a straightforward manner (Scheme 1). Activated Boc‐ValAlaPAB bromide **17** (Scheme S1) was used to capture the N‐terminal tertiary amine of tubulysin M through a quaternary ammonium linkage. The Boc group was removed from the dipeptide with TFA in dichloromethane followed by an allyl ester deprotection using palladium(0) with a pyrrolidine scavenger to provide intermediate **5**. Standard NHS coupling conditions were employed to install the self‐stabilizing mDPR maleimide,^[13]^ setting the stage for a final TFA deprotection to provide dipeptide drug‐linker **1**.

**Scheme 1:**
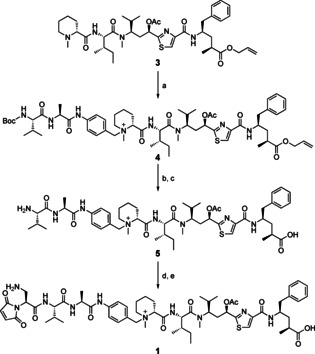
Synthesis of drug‐linker **1**. a) Boc‐ValAlaPAB−Br, butanone (86 %); b) TFA, CH_2_Cl_2_; c) Pd(PPh_3_)_4_, PPh_3_, pyrollidine, CH_2_Cl_2_ (80 %); d) mDPR(Boc)‐OSu, DIPEA, DMF (49 %); e) TFA, CH_2_Cl_2_ (54 %).

Concurrent to this effort, we hoped to apply the β‐glucuronidase^[14]^ cleavable glucuronide linker technology to the tubulysin drug class which we have shown can offset the liabilities associated with hydrophobic payloads such as ADC aggregation and accelerated clearance.^[15]^ The root of the synthetic challenge in incorporating the glucuronide linker was removing the glucuronide acetyl and acid protecting groups while leaving the tubuvaline acetate intact. Two tactics were employed that are unique to this synthesis as compared to the dipeptide work: 1) the free drug was incorporated into the drug‐linker in two parts by first quaternizing a protected *N*‐methyl pipecolic acid (**8**) residue followed by the introduction of the remaining tubulysin tripeptide through peptide coupling and 2) an unconventional titanium(IV) mediated transesterification was employed as a means to concurrently deprotect the glucuronide acetyl groups and transesterify the methyl ester to an orthogonal allyl ester while leaving the *tert*‐butyl Mep ester intact (Scheme 2).

**Scheme 2:**
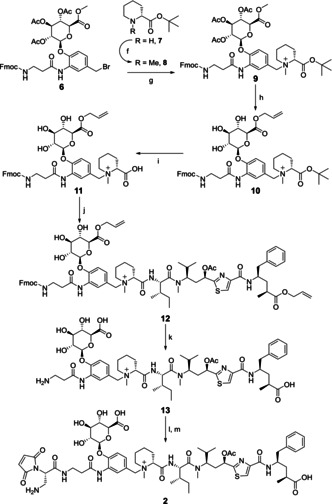
Synthesis of drug‐linker **2**. f) NaBH_3_CN, CH_2_O, AcOH, MeOH, H_2_O (96 %); g) butanone (82 %); h) Ti(OEt)_4_, allyl‐OH (48 %); i) TFA, CH_2_Cl_2_ (64 %); j) H‐IleTuvTup‐Oallyl (**18**), HATU, DIPEA, DMF (46 %); k) Pd(PPh_3_)_4_, PPh_3_, pyrollidine, CH_2_Cl_2_ (79 %); l) mDPR(Boc)‐OSu, DIPEA, DMF (33 %); m) TFA, CH_2_Cl_2_ (81 %).

Commercially available H‐Pip‐OtBu (**7**) was converted to Mep‐OtBu (**8**) via reductive amination and taken forward crude into a quaternization reaction with brominated glucuronide intermediate **6** (Scheme S2). The key transformation was performed in anhydrous allyl alcohol using Ti(OEt)_4,_ converting the methyl ester to allyl and removing the three acetyl groups from the glucuronic acid moiety. This product **9** was now positioned with excellent orthogonality to lead to our final drug‐linker. A TFA deprotection revealed the *N*‐methyl pipecolic acid which was coupled using HATU to the allyl‐protected tubulysin tripeptide **18** (Scheme S5). Palladium(0) was employed with a pyrrolidine scavenger to remove both allyl esters as well as a slow removal of the Fmoc group. This late intermediate **13** was coupled with mDPR(Boc)‐OSu, deprotected with TFA, and purified to provide glucuronide‐linked tubulysin M drug‐linker **2**.

Tubulysin drug‐linkers **1** and **2** were conjugated to an anti‐CD30 antibody as DAR 2 and mixed DAR 4 ADCs and evaluated for cytotoxicity against a panel of CD30+ lymphoma cell lines (Table 1). DAR 4 was selected as previous reports showed similar DAR 8 ADCs may suffer from sub‐optimal *in vivo* pharmacokinetic properties.[^7^, ^16^] The ADCs displayed nearly identical activities across the panel with IC_50_ values in the single‐digit to sub‐ng/mL range. Consistent with previous reports,[^10^, ^12b^] the tubulysin drug‐linkers maintained their activity against cell lines that displayed the MDR+ phenotype such as L428 and DELBVR. Ramos was included as a CD30‐negative control to confirm immunological specificity of the drug‐linkers; no cytotoxicity was observed at concentrations up to 1000 ng/mL. We engineered sites for conjugation at the 239 position on antibody heavy chains (S239C) to produce homogeneous DAR 2 ADCs.^[17]^ The *in vitro* activity of these engineered DAR 2 conjugates matched that of the DAR 4 ADCs on a drug molar basis against all cell lines tested (Table 1).

**Table 1:** ADC cytotoxicity, IC_50_s in ng/mL.^[a]^

ADC	DAR 4		DAR 2	
	αCD30‐**1**	αCD30‐**2**	αCD30‐**1**	αCD30‐**2**
L540cy *HL*	1.1 ng/mL	1.2	1.8	2.2
L428 *MDR+ HL*	0.3	0.3	0.9	0.8
DEL *ALCL*	0.4	0.5	1	1.2
DELBVR *MDR+ ALCL*	1.3	1.6	4	4.8
Ramos *CD30‐ NHL*	>1000	>1000	>1000	>1000

[a] Reported IC_50_ values are the average of at least two determinations.

In contrast to the equivalent activity observed *in vitro*, clear differences emerged in preclinical *in vivo* xenograft models. Mice implanted with CD30+ L540cy xenografts were treated with a single dose of αCD30‐**1** and αCD30‐**2** as DAR 4 ADCs (Figure 2A). The conjugate bearing the glucuronide linker **2** showed a significant improvement in activity over the dipeptide **1** comparator. Whereas the dipeptide achieved tumor growth‐delay and 2/5 cures at 2 mg/kg, the glucuronide achieved 5/5 cures at 0.6 mg/kg.

**Figure 2:**
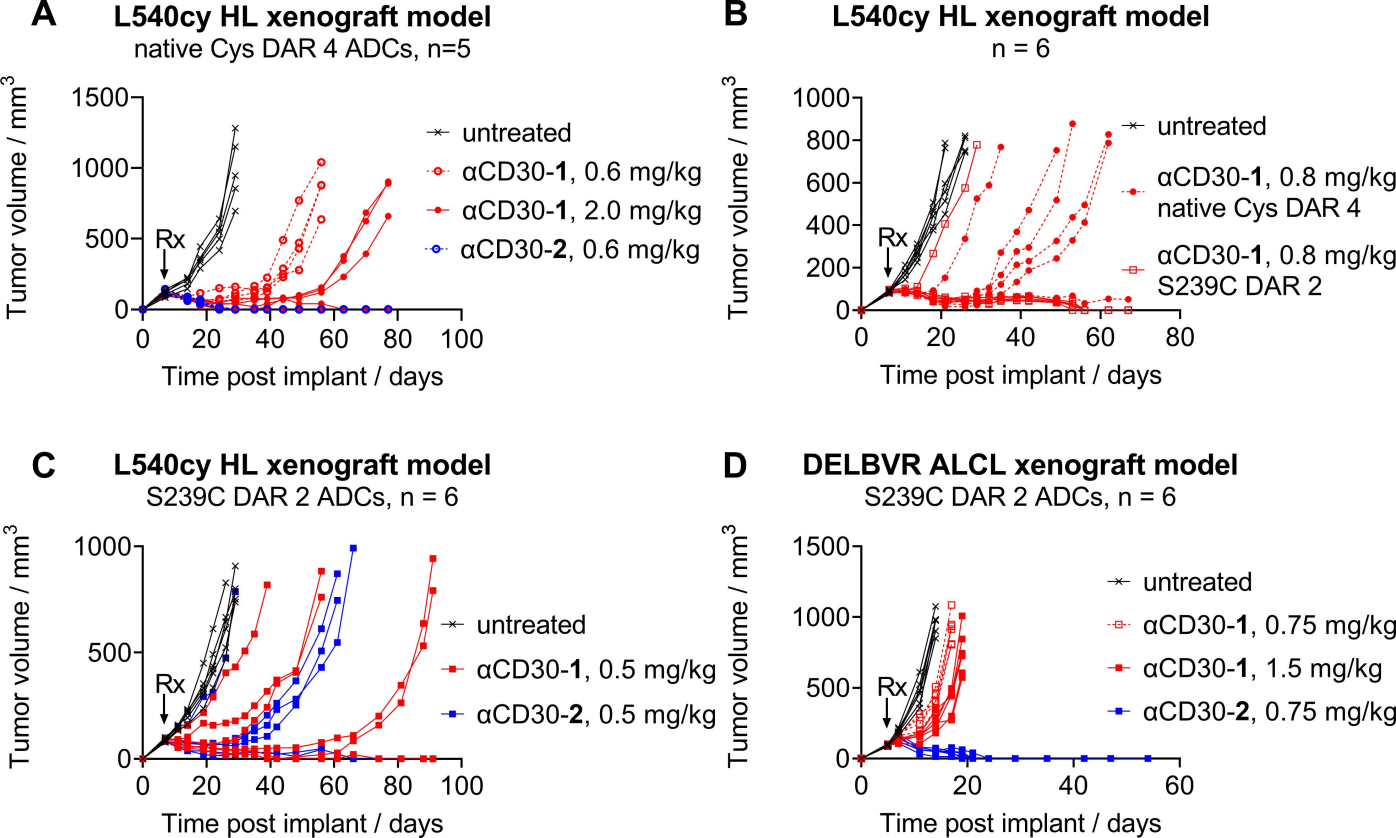
*In vivo* efficacy of αCD30 tubulysin ADCs administered as single‐dose IP injections. An L540cy Hodgkin lymphoma xenograft model was used to compare the antitumoral activity of A) DAR 4 ADCs bearing dipeptide linker **1** and glucuronide linker **2**, B) DAR 2 and DAR 4 ADCs bearing dipeptide linker **1**, and C) DAR 2 ADCs bearing dipeptide linker **1** and glucuronide linker **2**. D) A DELBVR ALCL xenograft model was used to further compare both linkers as DAR 2 ADCs

Site specific conjugation also had a striking impact on *in vivo* activity in L540cy xenografts (Figure 2B), in which a single 0.8 mg/kg dose of dipeptide **1** (DAR 4) conjugated to endogenous cysteines on cAC10 was compared to a DAR 2 ADC conjugated to S239C engineered cysteines. Despite halving the drug molar ratio, the DAR 2 ADCs led to 5/6 cures compared to 0/6 for the DAR 4 ADCs. These results taken together indicate that both the linker chemistry and conjugation site can lead to significant improvements in tubulysin ADC *in vivo* activity.

Considering the underperformance of the DAR 4 dipeptide ADCs in vivo, one area of concern was accelerated ADC clearance. To address this, total antibody plasma concentration was measured in a rat pharmacokinetic study to evaluate the relative clearance of dipeptide drug‐linker **1** as a DAR 4 and a DAR 8 ADC and glucuronide drug‐linker **2** as a DAR 8 ADC (Figure 3A). Both DAR 8 ADCs showed an increased rate of clearance compared to the unconjugated antibody. Consistent with past findings, the DAR 8 conjugates bearing the hydrophilic glucuronide linker provided increased exposure compared to those bearing dipeptide **1**.^[18]^ Changing to DAR 4 led to unimpaired pharmacokinetics for the more hydrophobic dipeptide **1** as compared to the unconjugated antibody indicating accelerated clearance was unlikely to be impacting DAR 4 ADC activity.

**Figure 3:**
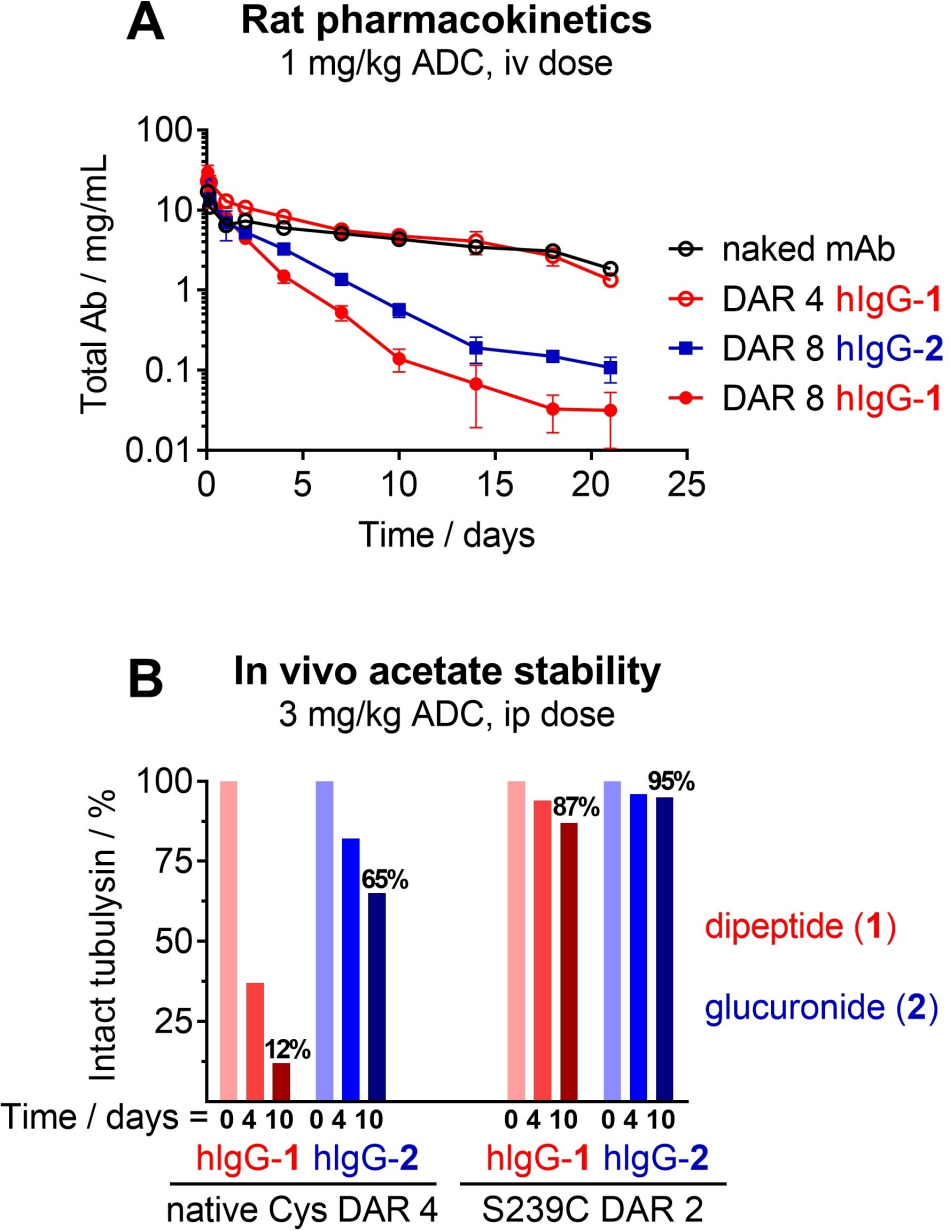
Tubulysin ADC *in vivo* pharmacokinetics and acetate stability. A) Circulating total antibody concentration in Sprague–Dawley rats of DAR 4 and DAR 8 ADCs bearing dipeptide **1** and DAR 8 ADCs bearing glucuronide **2** against an unconjugated antibody control ±SD. B) Percentage intact acetate in SCID mice monitored over time using PLRP‐MS for ADCs bearing dipeptide **1** or glucuronide **2** as DAR 2 and DAR 4 ADCs.

When comparing the dipeptide **1** and glucuronide **2** as DAR 2 site‐specific ADCs, the variability in activity between linkers appeared to decrease. In an L540cy xenograft (Figure 2C); both ADCs achieved 1/6 cures with a single dose of 0.5 mg/kg. Surprisingly, this equivalency was not consistent in a subsequent MDR+ DELBVR ALCL xenograft (Figure 2D). In this model the DAR 2 glucuronide **2** was highly active with 6/6 cures at 0.75 mg/kg, but the DAR 2 dipeptide **1** showed only slight tumor growth delay with no cures at twice the dose.

Acetate stability was evaluated *in vivo* for constructs **1** and **2** as DAR 4 endogenous cysteine conjugates and DAR 2 S239C engineered cysteine conjugates (Figure 3B). Blood draws taken from SCID mice at 4 and 10 days were analyzed by PLRP‐MS to determine the percent acetylated tubulysin. When conjugated to endogenous cysteines the dipeptide **1** and the glucuronide **2** showed stark differences; the DAR 4 dipeptide ADC had 12 % intact acetate after 10 days in circulation while the DAR 4 glucuronide ADC remained 65 % acetylated. Taken in conjunction with the previous unimpaired PK data of the DAR 4 dipeptide, this result is unlikely to be driven by differential clearance of the two linkers.

Conjugation to the S239C sites also provided acetate preservation. As a DAR 2 ADC the dipeptide **1** was 87 % intact after 10 days while the glucuronide **2** was 95 % intact. These results indicate that the glucuronide linker system, the S239C conjugation site, and particularly the combination significantly stabilize the C11 acetate in circulation.

The C11 acetate has long been identified as a potential liability in the tubulysin class and there have been several SAR efforts to replace it with stabilized moieties.[^9a^, ^10^, ^11^] Despite achieving potent free drug cytotoxicity, as ADC payloads these novel analogues have generally failed to match the *in vivo* efficacy of their acetylated progenitors.^[12]^ This gap in efficacy is most apparent in the context of MDR+ models, a potentially significant liability for a drug class that is distinct from many other microtubule inhibitors due to its ability to overcome resistance mechanisms.^[19]^ Staben et al. demonstrated the improved activity of a stabilized ether tubulysin ADC relative to an MMAE‐based control in an MDR+ model, but did not directly compare it to the analogous parent tubulysin.^[9b]^ In the context of this earlier work we sought to utilize the unique opportunities afforded by ADC design to offset the liabilities of the C11 acetate without the need to replace it.

Through a strategic Ti^IV^‐mediated transesterification we were able to combine our quaternary ammonium linker technology^[7]^ with the hydrophilic glucuronide release trigger^[15]^ to link and release unmodified tubulysin M while improving the stability of the C11 acetate. This stability increase led to improved activity in mouse xenograft models and represents a unique finding regarding the ability of the glucuronide linker to directly impact payload stability. Total removal of an enzymatically cleavable spacer has been shown to have a stabilizing effect on tubulysin ADCs,^[20]^ but to our knowledge this is the first example where a conversion to an alternative enzymatically cleavable drug‐linker preserves a labile payload in circulation. This finding could have utility extending to other labile drug classes beyond tubulysin that benefit from such a cleavable sequence.

Site specific conjugation has already been shown to have an impact on ADC stability regarding maleimide transfer,^[17]^ and earlier work has shown this is applicable to the tubulysin acetate as well.[^12a^, ^20^] Dipeptide **1** linked here via the sheltered S239C site outperformed their endogenous cysteine linked comparators, often more than doubling the *in vivo* efficacy of the same payload. That the same improvement was *not* observed when linking glucuronide **2** to S239C supports the hypothesis that both the glucuronide and S239C site improve activity via the same mechanism: preservation of the C11 acetate.

Although S239C conjugation generally led to similar ADC activity between the glucuronide and dipeptide linked conjugates, this proved not to be true in every indication. The superior activity of the glucuronide linked ADC in a DELBVR xenograft model could be a result of the underlying biology of the ALCL model, or a differentiating property of the release mechanism of the glucuronide linker. It is a surprising finding and will be investigated further.

ADCs are complex therapeutics, often greater than the sum of their parts. This work demonstrates how pairing innovations in antibody engineering and drug‐linker design can optimize the targeted delivery of a promising payload. The resulting glucuronide‐tubulysin ADCs are stable, specific, homogenous, and provide a unique activity profile to enable future ADC programs.

## Conflict of interest

The authors declare no conflict of interest.

## Supporting information

As a service to our authors and readers, this journal provides supporting information supplied by the authors. Such materials are peer reviewed and may be re‐organized for online delivery, but are not copy‐edited or typeset. Technical support issues arising from supporting information (other than missing files) should be addressed to the authors.

Supplementary
