# Supplementary material for: Improving Antibody‐Tubulysin Conjugates through Linker Chemistry and Site‐Specific Conjugation
Source: ChemMedChem. 2021 Feb 12;16(7):1077–81. doi: 10.1002/cmdc.202000889 (PMC8048973; doi:10.1002/cmdc.202000889)
Supplement: Supplementary file 1 — Supplementary [file CMDC-16-1077-s001.pdf]

ChemMedChem

Supporting Information

Improving Antibody-Tubulysin Conjugates through Linker Chemistry and Site-Specific Conjugation

Joseph Z. Hamilton,* Thomas A. Pires, Jamie A. Mitchell, Julia H. Cochran, Kim K. Emmerton, Margo Zaval, Ivan J. Stone, Martha E. Anderson, Steven Jin, Andrew B. Waight, Robert P. Lyon, Peter D. Senter, Scott C. Jeffrey, and Patrick J. Burke

Supplemental Information:

Supplemental Table 1. Tubulysin free drug in vitro cytotoxicity

Free Drug Cytotoxicity

Drug	L540cy IC_{50} HL	L428 IC_{50} HL	HEL92.1.7 IC_{50} AML	786-O IC_{50} RCC
**tubulysin M**	**0.1 nM**	**0.07**	**0.3**	**0.2**
**deacetylated tubulysin M**	**19**	**62**	**43**	**101**

ADC Conjugation

To achieve partial reduction of interchain disulfides, antibody in PBS solution was bound to a MabSelectSuRe protein A column and incubated with a 2.2-3 equivalents of tris(2-carboxyethyl)-phosphine (TCEP) for 30 minutes. The column was then washed thoroughly with PBS containing 5 mmol/L EDTA. The reduced mAb was eluted with 50 mmol/L glycine (pH 3.0), and the eluate was neutralized with an addition of concentrated sodium phosphate buffer to a final formulation of 80 mmol/L sodium phosphate, 50 mmol/L NaCl, 45 mmol/L glycine, 5 mmol/L EDTA, pH 7.4. Antibody at approximately 10 mg/mL was conjugated with ~25% excess of drug-linker as a 10 mmol/L DMSO stock. The resulting solution was vortexed and left at room temperature for 15 to 30 minutes. The extent of conjugation was assessed by PLRP-UV, and additional drug-linker was added as needed. Once all available mAb cysteines were alkylated, excess drug-linker was removed via incubation with activated charcoal. The ADC was then buffer-exchanged into PBS using a NAP-5 desalting column (GE Healthcare). The extent of aggregation was assessed by size exclusion chromatography and in all cases monomeric ADCs were obtained. The final ADC concentration was measured spectrophotometrically, and the resulting ADCs were sterile-filtered through a 0.22-mm centrifugal filter and stored at -80 °C.

To prepare reduced S239C engineered cysteine antibodies for conjugation, the above method was followed using a large excess of TCEP for full reduction of the antibody. After reduction of the engineered cysteine antibody, interchain disulfides were re-oxidized by the addition of 7 equivalents of dehydroascorbic acid for 1 hour at room temperature, followed by the addition of 10 eq dehydroascorbic acid for another 1-hour incubation. Each reduced antibody was confirmed by reversed-phase chromatography (PLRP 3 μm, Agilent) by analyzing a small aliquot conjugated to MC-VC-MMAE.

In vitro cytotoxicity assays

L540cy (Hodgkin lymphoma, HL) was provided by Dr. Harald Stein (Institute fur Pathologie, University of Veinikum Benjamin Franklin, Berlin, Germany). L428 (HL) and DEL (anaplastic large cell lymphoma (ALCL)) were obtained from DSMZ. Ramos (non-Hodgkin lymphoma, NHL), HEL92.1.7 (acute myeloid leukemia, AML) and 786-O (renal cell carcinoma, RCC) were obtained from ATCC. The cell lines were authenticated by STR profiling at IDEXX Bioresearch and cultured for no more than 2 months after resuscitation. Cells cultured in log-phase growth were seeded for 24 hours in 96-well plates containing 150 mL RPMI 1640 supplemented with 20% FBS. Serial dilutions of antibody–drug conjugates in cell culture media were prepared at 4× working concentrations, and 50 mL of each dilution was added to the 96-well plates. Following addition of test articles, cells were incubated with test articles for 4 days at 37°C. After 96 hours, growth inhibition was assessed by CellTiter-Glo (Promega), and luminescence was measured on a plate reader. The IC_{50} value, determined in triplicate, is defined here as the concentration that results in 50% reduction in cell growth relative to untreated controls.

Tubulin fluorescence polarization competition-binding assay

Sheep brain tubulin (SBT) was obtained from Cytoskeleton, and exact protein concentration was determined using the DC protein assay (Bio-Rad Laboratories). Eight-point serial dilutions of test

compounds were performed in assay buffer (20 mmol/L PIPES, pH 6.9, 1 mmol/L EGTA + 0.008% Tween 20) + 60 nmol/L FITC-MMAF probe for competition representing a 2× assay concentration (highest amount 10 μmol/L and dilutions occurring at 5× concentration). To initiate the assay, 15 mL of (2×) test compound dilution + 60 nmol/L FITC-MMAF was combined with 15 ml (2×) 600 nmol/L SBT in assay buffer in the wells of a 384-well plate for a final concentration of 30 nmol/L FITC-MMAF, 300 nmol/L SBT, and 8 test compound concentration points performed in duplicate. The plate was covered, and the binding competition was allowed to proceed for 1 hour at room temperature with gentle shaking. Fluorescence polarization was measured on an Envision multilabel reader (Perkin Elmer) using an installed FITC FP dual mirror. Measurements of polarization (milli-polarization units) are defined as (mP) = 1,000 (S - G × P)/(S + G × P), where S and P represent the parallel and perpendicular background subtracted fluorescence count rates following polarized excitation, and G (grating) is an instrument-dependent factor calculated from pure fluorophore solution. Binding data were analyzed using GraphPad Prism software.

In vivo xenograft studies

All in vivo experiments were reviewed and approved by the Seagen Institutional Animal Care and Use Committee in a facility fully accredited by the Association for Assessment and Accreditation of Laboratory Animal Care (AAALAC). Accreditation numbers were not available nor needed to proceed with the studies. Immunocompromised SCID mice bearing L540cy or DELBVR xenografts were administered a single I.P. dose upon tumor volume reaching 100 mm^3. Tumor volume was determined using the formula $(L \times W^2)/2$. Animal were euthanized when tumors reached 1000 mm^3.

In vivo rat pharmacokinetics

Pharmacokinetic experiments were performed using radiolabeled antibody or ADC. To a solution of mAb or ADC in PBS supplemented with an additional 50 mmol/L potassium phosphate (pH 8.0) and 50 mmol/L sodium chloride was added 55 mCi N10 succinimidyl propionate, [propionate-2,3-3H]- (Moravek Biochemicals 80 Ci/mmol, 1 mCi/mL, 9:1 hexane:ethyl acetate solution) per mg of antibody or ADC. The resulting solution was vortexed and left at room temperature for 2 hours, centrifuged at 4,000 g for 5 minutes, and the lower aqueous layer was removed and split into 30-kDa MWCO Amicon Ultra-15 Centrifugal Filter Units (Millipore). Unconjugated radioactivity was removed by four rounds of dilution and centrifugation at 4,000 g. The resulting products were filtered through sterile 0.22 mm Ultrafree-MC Centrifugal Filter Units (Millipore) and the final antibody or ADC concentration was measured spectrophotometrically. The specific activity (mCi/mg) of each product was determined by liquid scintillation counting. 1 mg of radiolabeled antibody or ADC per kg of animal weight was injected via the tail vein. Each test article was dosed once in replicate animals. Blood was drawn into K2EDTA tubes via the saphenous vein at various time points. Plasma was isolated by centrifugation for 10 minutes at 10,000 g. A 10- to 20-mL sample of plasma from each time point was added to 4 mL Ecoscint-A liquid scintillation cocktail (National Diagnostics), and the total radioactivity was measured by liquid scintillation counting. The resulting disintegrations per minute values were converted to mCi and the specific activity of the radiolabeled test articles was used to calculate the mAb or ADC concentrations remaining in the plasma at each time point.

In vivo acetate stability

SCID mice were administered conjugate as a single I.P. dose of 3 mg/kg and then subjected to terminal bleeds at 4- and 10-days post-dose. Blood samples from each animal were processed to plasma using centrifugation into EDTA coated Eppendorf tubes. The plasma was batch purified using anti-human capture affinity resin (IgSelect, GE Healthcare) for two hours at 2-8 °C. The bound samples were washed using 0.5 M NaCl and eluted using 50 mM glycine, pH 3. Eluted samples were deglycosylated using PNGase F (New England BioLabs Inc) then reduced using 5 mM DTT. Each sample was analyzed using reversed-phased UPLC (PLRP xum, Agilent) coupled with mass spectrometric detection (Waters Xevo G2-S QTOF). Intact drug (% acetylation) was calculated using total ion counts of the drug loaded light chain and heavy chain species, assessed by a loss of 42 daltons.

Chemistry Experimentals:

General Information:

All commercially available anhydrous solvents were used without further purification. Column chromatography was performed on a Biotage Isolera One flash purification system (Charlotte, NC). UPLC-MS system 1 consisted of a Waters SQ mass detector interfaced to an Acquity Ultra Performance LC equipped with an Acquity UPLC BEH C18 2.1 x 50 mm, 1.7μm reverse phase column. The acidic mobile phase (0.1% formic acid) consisted of a gradient of 3% acetonitrile/97% water to 100% acetonitrile (flow rate = 0.5 mL/min). UPLC-MS system 2 consisted of a Waters Xevo G2 ToF mass spectrometer interfaced to a Waters Acquity H-Class Ultra Performance LC equipped with an Acquity UPLC BEH C18 2.1 x 50 mm, 1.7μm reverse phase column. The acidic mobile phase (0.1% formic acid) consisted of a gradient of 3% acetonitrile/97% water to 100% acetonitrile (flow rate = 0.7 mL/min). Preparative HPLC was carried out using a Waters Prep 150 LC system paired with a 2998 photodiode array detector. Products were purified over a C12 Phenomenex Synergi 10.0 x 250 mm, 4 μm, 80 Å reverse phase column eluting with 0.1% trifluoroacetic acid in water (solvent A) and 0.1% trifluoroacetic acid in acetonitrile (solvent B). The purification methods generally consisted of linear gradients of solvent A to solvent B, ramping from 90% aqueous solvent A to 10% solvent A over 1 hour. The flow rate was 4.6 mL/min with monitoring at 254 nm. ^{1}H NMR spectra were collected on a Varian 400 MHz, a Bruker 300 MHz, or a Varian 500 MHz instrument as indicated.

Supplemental Scheme 1:

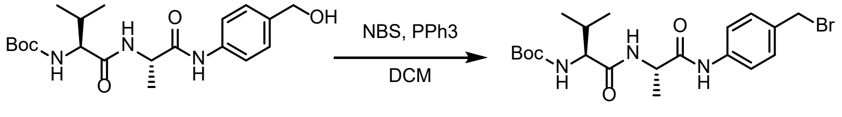

16 17

tert-butyl ((S)-1-(((S)-1-((4-(bromomethyl)phenyl)amino)-1-oxopropan-2-yl)amino)-3-methyl-1-oxobutan-2-yl)carbamate (17): A flask containing Boc-Val-Ala-PABA-OH ((**16**), 100 mg, 254 μmol), N-bromosuccinimide (68 mg, 381 μmol), and triphenylphosphine (100 mg, 381 μmol) was flushed with nitrogen. The reaction was taken up in DCM (4 mL) and stirred for 12 hours. The reaction was condensed and purified over silica via a Biotage column (Hexanes/EtOAc, 10%-100%) to provide **17** (94 mg, 81%). Analytical UPLC-MS (system 1): tr=2.09 min, m/z (ES+) calculated 456.15 $[M+H]^+$; found 456.10. ^{1}H NMR (400 MHz, DMF-d_7) δ 8.74 (d, *J* = 4.8 Hz, 1H), 8.43 (d, *J* = 6.9 Hz, 1H), 7.96 – 7.82 (m, 2H), 7.64 – 7.56 (m, 1H), 7.60 – 7.41 (m, 1H), 6.87 (d, *J* = 8.4 Hz, 1H), 4.88 (s, 1H), 4.85 – 4.69 (m, 1H), 4.20 (dd, *J* = 8.5, 6.1 Hz, 1H), 3.92 (s, 4H), 2.30 (dt, *J* = 13.4, 6.8 Hz, 1H), 1.61 (dd, *J* = 16.0, 7.1 Hz, 3H), 1.56 (s, 6H), 1.28 (dd, *J* = 6.9, 3.5 Hz, 2H), 1.11 (dd, *J* = 17.8, 6.8 Hz, 4H).

Supplemental Scheme 2:

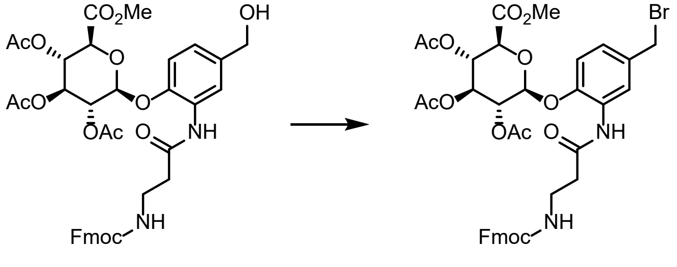

15 6

(2S,3R,4S,5S,6S)-2-(2-(3-((((9H-fluoren-9-yl)methoxy)carbonyl)amino)propanamido)-4-(bromomethyl)phenoxy)-6-(methoxycarbonyl)tetrahydro-2H-pyran-3,4,5-triyl triacetate (6): A flame-

dried flask was charged with known glucuronide linker fragment[1] (**15**, 210 mg, 281 μmol) in 4.5 mL anhydrous THF. The solution was stirred at room temperature under nitrogen. Triphenylphosphine (111 mg, 421.5 μmol) and N-bromosuccinimide (75 mg, 421.5 μmol) were added sequentially and the solution was stirred for 2 hours. The reaction was condensed under reduced pressure and purified over silica via a Biotage column (Hexanes/EtOAc, 30%-50%-70%) to provide **6** (222 mg, 97%). Analytical UPLC-MS (system 1): tr = 2.36 min, m/z (ES+) calculated 811.17 $[M+H]^+$; found 811.34. 1H NMR (400 MHz, Chloroform-d) δ 8.48 (s, 1H), 8.05 (s, 1H), 7.76 (d, J = 7.5 Hz, 2H), 7.60 (d, J = 7.5 Hz, 2H), 7.39 (t, J = 7.4 Hz, 2H), 7.30 (t, J = 7.8 Hz, 2H), 7.26 (s, 1H), 7.09 (dd, J = 8.4, 2.2 Hz, 1H), 6.91 (d, J = 8.4 Hz, 1H), 5.64 (s, 1H), 5.46 – 5.37 (m, 1H), 5.30 (q, J = 9.6, 8.9 Hz, 2H), 5.05 (d, J = 7.5 Hz, 1H), 4.47 (s, 2H), 4.45 – 4.31 (m, 1H), 4.27 – 4.07 (m, 2H), 3.74 (d, J = 0.5 Hz, 3H), 3.60 (d, J = 6.1 Hz, 2H), 2.80 – 2.70 (m, 3H), 2.09 – 2.03 (m, 9H), 1.26 (td, J = 7.1, 0.6 Hz, 1H).

Supplemental Scheme 3:

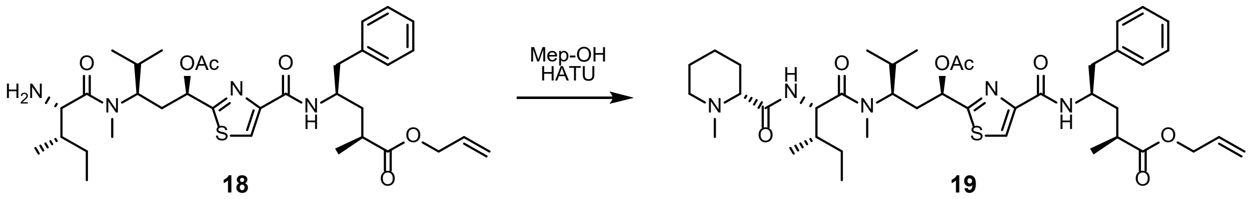

allyl (2S,4R)-4-(2-((1R,3R)-1-acetoxy-3-((2S,3S)-N,3-dimethyl-2-((R)-1-methylpiperidine-2-carboxamido)pentanamido)-4-methylpentyl)thiazole-4-carboxamido)-2-methyl-5-phenylpentanoate (19): (R)-N-methyl pipecolic acid (AK Scientific, 22 mg, 153 μmol) and HATU (53 mg, 140 μmol) were dissolved in anhydrous DMF (0.64 mL). DIPEA (89 μL, 510 μmol) was added and the reaction was stirred for 10 minutes. Tripeptide **18** was dissolved in an additional 0.64 mL DMF and added to the reaction in solution. LCMS indicated full conversion after 5 minutes. The reaction was condensed under reduced pressure and purified by preparative LC to provide **19** (69 mg, 71%). Analytical UPLC-MS (system 2): t_r = 1.59 min, *m/z* (ES+) calculated 768.44 $[M+H]^+$; found 768.46. 1H NMR (400 MHz, Methanol-d4) δ 8.07 (s, 1H), 7.28 – 7.12 (m, 5H), 5.85 (m, J = 16.3, 10.8, 5.6 Hz, 1H), 5.70 (dd, J = 11.0, 2.7 Hz, 1H), 5.47 (s, 1H), 5.23 (dq, J = 17.2, 1.8 Hz, 1H), 5.18 – 5.10 (m, 1H), 4.73 (d, J = 7.9 Hz, 3H), 4.56 – 4.42 (m, 3H), 4.42 – 4.31 (m, 1H), 3.23 (d, J = 12.3 Hz, 3H), 3.10 (s, 4H), 2.88 (qd, J = 13.6, 6.8 Hz, 2H), 2.68 – 2.57 (m, 1H), 2.50 (s, 3H), 2.43 – 2.31 (m, 1H), 2.31 – 2.17 (m, 1H), 2.14 (s, 3H), 2.05 – 1.94 (m, 2H), 1.87 (s, 2H), 1.83 (s, 2H), 1.81 – 1.70 (m, 1H), 1.73 – 1.55 (m, 2H), 1.51 – 1.42 (m, 0H), 1.26 – 1.16 (m, 1H), 1.16 (d, J = 7.1 Hz, 4H), 1.05 – 0.87 (m, 8H), 0.82 (d, J = 6.6 Hz, 3H).

Supplemental Scheme 4:

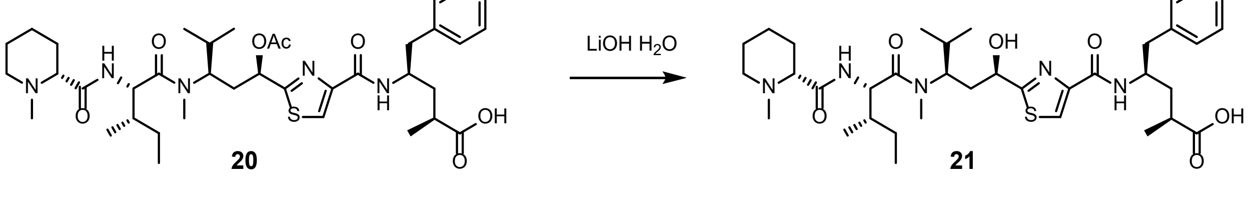

(2S,4R)-4-(2-((1R,3R)-3-((2S,3S)-N,3-dimethyl-2-((R)-1-methylpiperidine-2-carboxamido)pentanamido)-1-hydroxy-4-methylpentyl)thiazole-4-carboxamido)-2-methyl-5-phenylpentanoic acid (21): A flask charged with tubulysin M (Levena Biopharma, 6.0 mg, 8 μmol), MeOH (0.42 mL), and THF (0.42 mL) was cooled to 0° C while stirring. LiOH · H_2O (1.4 mg, 33 μmol) was dissolved in H_2O (0.42 mL) and added dropwise. The reaction was allowed to warm to room temperature,

the LCMS indicated full conversion after 1.5 hours. The reaction was quenched with acetic acid (1.9 µL 33 µmol) and condensed under reduced pressure. The residue was taken up in minimal DMSO and purified by preparative LC to provide **21** (6 mg, 106%). Analytical UPLC-MS: t_r = 1.05 min, *m/z* (ES+) calculated 686.40 $[M+H]^+$; found 686.59.

Supplemental Scheme 5:

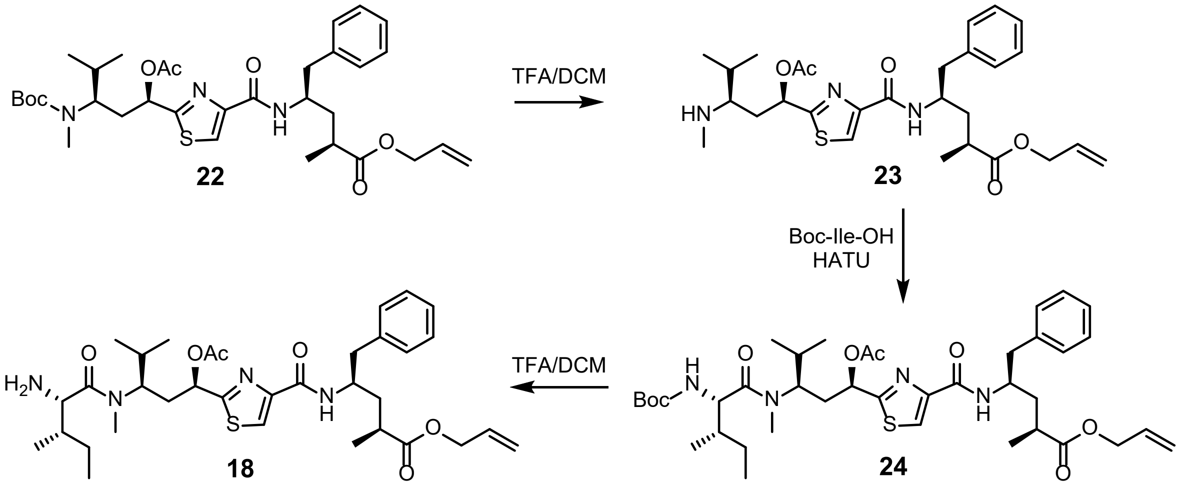

allyl (2S,4R)-4-(2-((1R,3R)-1-acetoxy-4-methyl-3-(methylamino)pentyl)thiazole-4-carboxamido)-2-methyl-5-phenylpentanoate (23): A flask charged with Boc-Tuv(OAc)-Tup-OAllyl[2] (**22,** 33 mg, 21 µmol) was cooled to 0 °C under N_2. A solution of 10% TFA in CH_2Cl_2 (0.52 mL) was added dropwise and stirred for 4 hours. The reaction was concentrated under reduced pressure, resolubilized in DCM, and condensed 3 times to remove TFA then carried forward without further purification. Analytical UPLC-MS (system 2): t_r = 1.03 min, *m/z* (ES+) calculated 530.27 $[M+H]^+$; found 530.36. 1H NMR (500 MHz, Chloroform-d) δ 8.26 (s, 1H), 7.96 (s, 1H), 7.66 (d, J = 9.5 Hz, 1H), 7.31 – 7.23 (m, 2H), 7.23 – 7.10 (m, 3H), 6.22 (dd, J = 8.5, 4.6 Hz, 1H), 5.84 (ddt, J = 17.3, 10.4, 5.8 Hz, 1H), 5.32 – 5.22 (m, 1H), 5.20 (dq, J = 10.5, 1.3 Hz, 1H), 4.54 (qdt, J = 13.2, 5.8, 1.4 Hz, 2H), 4.43 (tdd, J = 10.4, 6.8, 3.5 Hz, 1H), 3.05 (q, J = 7.3, 5.4 Hz, 1H), 2.94 (dd, J = 13.9, 6.8 Hz, 1H), 2.86 (dd, J = 13.9, 7.2 Hz, 1H), 2.81 (t, J = 5.6 Hz, 3H), 2.63 (dqd, J = 10.9, 7.1, 3.9 Hz, 1H), 2.42 (ddd, J = 15.8, 8.9, 4.7 Hz, 1H), 2.34 – 2.17 (m, 2H), 2.24 (s, 3H), 2.04 (ddd, J = 14.1, 10.3, 3.6 Hz, 1H), 1.65 (ddd, J = 14.4, 10.5, 3.9 Hz, 1H), 1.18 (d, J = 7.1 Hz, 3H), 1.05 (dd, J = 16.9, 6.9 Hz, 6H).

allyl (2S,4R)-4-(2-((6S,9R,11R)-6-((S)-sec-butyl)-9-isopropyl-2,2,8-trimethyl-4,7,13-trioxo-3,12-dioxa-5,8-diazatetradecan-11-yl)thiazole-4-carboxamido)-2-methyl-5-phenylpentanoate (24): To a flask charged with H-Tuv(OAc)-Tup-OAllyl (**23**, 28 mg, 53 µmol) was added Boc-Ile-OH (15 mg, 63 µmol) and HATU (40 mg, 106 µmol) as solids followed by DMF (1.0 mL). N,N-Diisopropylethylamine (37 uL, 211 µmol) was added and the reaction was stirred at room temperature for 48 hours. The reaction was then taken up in DMSO, condensed under reduced pressure, and purified by preparative LC to provide **24** (19 mg, 49%). Analytical UPLC-MS (system 2): t_r = 1.72 min, *m/z* (ES+) calculated 743.41 $[M+H]^+$; found 743.51.

allyl (2S,4R)-4-(2-((1R,3R)-1-acetoxy-3-((2S,3S)-2-amino-N,3-dimethylpentanamido)-4-methylpentyl)thiazole-4-carboxamido)-2-methyl-5-phenylpentanoate (18): A flask charged with Boc-Ile-Tuv(OAc)-Tup-OEt (**24**, 19 mg, 26 µmol) was cooled to 0 °C under N_2. A solution of 10% TFA in CH_2Cl_2 (0.52 mL) was added dropwise and stirred for 4 hours. The reaction was concentrated under reduced pressure, resolubilized in DCM, and condensed 3 times to remove TFA then carried forward without further purification. Analytical UPLC-MS (system 2): t_r = 1.18 min, *m/z* (ES+) calculated 643.36 $[M+H]^+$; found 643.42. 1H NMR (500 MHz, DMSO-d6) δ 8.17 (s, 1H), 7.94 (d, J = 9.1 Hz, 1H), 7.26 – 7.19 (m, 3H), 7.19 – 7.11 (m, 3H), 6.02 (s, 2H), 5.81 (ddt, J = 17.3, 10.7, 5.4 Hz, 1H), 5.50 (dd, J = 11.1,

2.5 Hz, 1H), 5.20 (dq, J = 17.3, 1.7 Hz, 1H), 5.12 (dq, J = 10.5, 1.5 Hz, 1H), 4.49 – 4.36 (m, 2H), 4.29 (s, 1H), 4.26 – 4.15 (m, 2H), 2.89 (s, 3H), 2.85 (dd, J = 13.6, 7.2 Hz, 2H), 2.76 (dd, J = 13.6, 6.6 Hz, 1H), 2.52 – 2.45 (m, 1H), 2.35 – 2.25 (m, 1H), 2.18 (t, J = 13.3 Hz, 1H), 2.07 (s, 3H), 1.88 – 1.74 (m, 2H), 1.65 (ddd, J = 14.2, 10.1, 4.5 Hz, 1H), 1.49 – 1.38 (m, 1H), 1.11 – 0.97 (m, 6H), 0.96 (d, J = 6.5 Hz, 3H), 0.85 (t, J = 7.3 Hz, 3H), 0.77 (d, J = 6.6 Hz, 3H).

Supplemental Scheme 6:

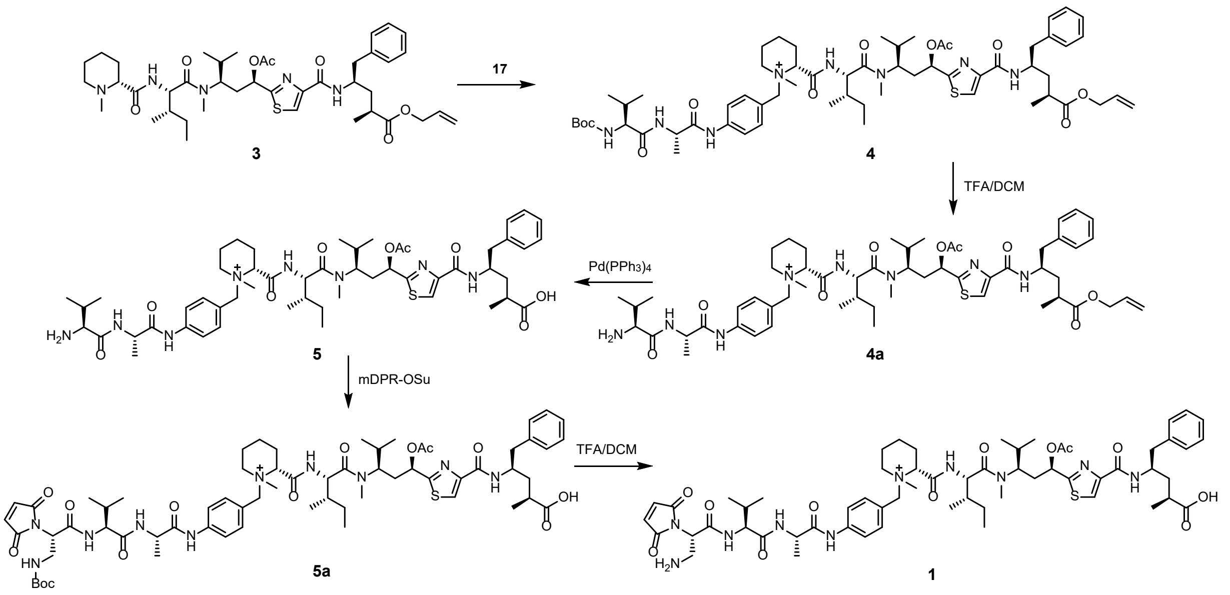

(2R)-2-(((2S,3S)-1-(((1R,3R)-1-acetoxy-1-(4-(((2R,4S)-5-(allyloxy)-4-methyl-5-oxo-1-phenylpentan-2-yl)carbamoyl)thiazol-2-yl)-4-methylpentan-3-yl)(methyl)amino)-3-methyl-1-oxopentan-2-yl)carbamoyl)-1-(4-((S)-2-((S)-2-((tert-butoxycarbonyl)amino)-3-methylbutanamido)propanamido)benzyl)-1-methylpiperidin-1-ium (4): A pressure vessel was charged with Boc-Val-Ala-PAB-Br (**17**, 59 mg, 130 μmol) and Tub-OAllyl (**19**, 50 mg, 65 μmol) in anhydrous 2-butanone (1.3 mL). The reaction was flushed with N_2, sealed, and allowed to stir at 80 ^{O}C for 12 hours. The reaction was condensed, taken up in DMSO, and purified by preparative LC to yield **4** (64 mg, **86%**). Analytical UPLC-MS (system 2): t_r = 1.39 min, *m/z* (ES+) calculated 1143.65 $[M]^+$; found 1143.78.

(2R)-2-(((2S,3S)-1-(((1R,3R)-1-acetoxy-1-(4-(((2R,4S)-5-(allyloxy)-4-methyl-5-oxo-1-phenylpentan-2-yl)carbamoyl)thiazol-2-yl)-4-methylpentan-3-yl)(methyl)amino)-3-methyl-1-oxopentan-2-yl)carbamoyl)-1-(4-((S)-2-((S)-2-amino-3-methylbutanamido)propanamido)benzyl)-1-methylpiperidin-1-ium (4a): : A flask charged with Boc-Val-Ala-PABQ-Tub-OAllyl (**4**, 50 mg, 44 μmol) in anhydrous DCM (1.75 mL) was cooled to 0C while stirring. TFA (0.44 mL) was added dropwise and the reaction was allowed to warm to room temperature. LCMS showed full removal of the Boc group after 1.5 hours at which point the reaction was condensed under reduced pressure, dried under vacuum overnight, and carried forward without further purification. Analytical UPLC-MS (system 2): t_r = 1.13 min, *m/z* (ES+) calculated 1043.60 $[M]^+$; found 1043.74.

(2R)-2-(((2S,3S)-1-(((1R,3R)-1-acetoxy-1-(4-(((2R,4S)-4-carboxy-1-phenylpentan-2-yl)carbamoyl)thiazol-2-yl)-4-methylpentan-3-yl)(methyl)amino)-3-methyl-1-oxopentan-2-yl)carbamoyl)-1-(4-((S)-2-((S)-2-amino-3-methylbutanamido)propanamido)benzyl)-1-methylpiperidin-1-ium (5): Crude H-TuvTup-OAllyl (57 mg, 55 μmol) was resolubilized in anhydrous DCM (2.7 mL) followed by the addition of pyrollidine (36 μL, 437 μmol), $Pd(PPh_3)_4$ (6.3 mg, 5.5 μmol), and PPh_3 (2.9 mg, 10.9 μmol). LCMS indicated full removal of the allyl ester after 1 hour. The reaction was diluted in DMSO, condensed under reduced pressure, then purified by preparative LC to provide **5** (44

mg, 80%). Analytical UPLC-MS (system 2): t_r = 1.01 min, *m/z* (ES+) calculated 1003.57 $[M]^+$; found 1003.70.

(2R)-2-(((2S,3S)-1-(((1R,3R)-1-acetoxy-1-(4-(((2R,4S)-4-carboxy-1-phenylpentan-2-yl)carbamoyl)thiazol-2-yl)-4-methylpentan-3-yl)(methyl)amino)-3-methyl-1-oxopentan-2-yl)carbamoyl)-1-(4-((7S,10S,13S)-7-(2,5-dioxo-2,5-dihydro-1H-pyrrol-1-yl)-10-isopropyl-2,2,13-trimethyl-4,8,11-trioxo-3-oxa-5,9,12-triazatetradecan-14-amido)benzyl)-1-methylpiperidin-1-ium (5a): mDPR-OSu (1.3 mg, 3.5 μmol) was taken up in DMF (0.3 mL) and added to a flask containing Val-Ala-PAB-TubM (**5**, 3.2 mg, 3.2 μmol). N,N-diisopropylethylamine (1.6 mg, 13 μmol) was added and the reaction was stirred under N_2 for 3 hours. The reaction was taken up in DMSO and purified by preparative LC to yield **5a** (2.0 mg, **49%**). Analytical UPLC-MS (system 2): t_r = 1.35 min, *m/z* (ES+) calculated 1269.66 $[M]^+$; found 1269.76.

(2R)-2-(((2S,3S)-1-(((1R,3R)-1-acetoxy-1-(4-(((2R,4S)-4-carboxy-1-phenylpentan-2-yl)carbamoyl)thiazol-2-yl)-4-methylpentan-3-yl)(methyl)amino)-3-methyl-1-oxopentan-2-yl)carbamoyl)-1-(4-((S)-2-((S)-2-((S)-3-amino-2-(2,5-dioxo-2,5-dihydro-1H-pyrrol-1-yl)propanamido)-3-methylbutanamido)propanamido)benzyl)-1-methylpiperidin-1-ium (1): A flask containing mDPR(Boc)-Val-Ala-PAB-TubM (**5a**, 2 mg, 1.6 μmol) was cooled to 0 °C under N_2. A solution of 10% TFA in CH_2Cl_2 (1.6 mL) was added dropwise and stirred for 4 hours. The reaction was condensed, taken up in DMSO, and purified by preparative LC to yield **1** (1.0 mg, **54%**). Analytical UPLC-MS (system 2): t_r = 1.02 min, *m/z* (ES+) calculated 1169.61 $[M]^+$; found 1169.72. ^{1}H NMR (400 MHz, Acetonitrile-d_3) δ 8.90 (s, 1H), 8.00 (s, 6H), 7.86 (s, 1H), 7.61 (s, 1H), 7.55 (d, *J* = 8.3 Hz, 2H), 7.42 (d, *J* = 9.4 Hz, 1H), 7.19 (d, *J* = 7.5 Hz, 1H), 7.18 – 7.08 (m, 4H), 7.04 (d, *J* = 8.0 Hz, 1H), 6.75 (d, *J* = 6.6 Hz, 2H), 5.69 (dt, *J* = 13.3, 6.7 Hz, 1H), 5.14 (t, *J* = 6.6 Hz, 1H), 4.64 (q, *J* = 7.4 Hz, 1H), 4.47 (s, 1H), 4.37 (s, 1H), 4.24 (t, *J* = 9.2 Hz, 2H), 4.07 (s, 1H), 3.90 (dt, *J* = 11.4, 6.4 Hz, 1H), 3.67 – 3.59 (m, 1H), 3.55 (s, 1H), 3.04 (s, 2H), 2.88 (s, 3H), 2.79 (d, *J* = 6.9 Hz, 2H), 2.72 (s, 2H), 2.46 – 2.38 (m, 1H), 2.24 (t, *J* = 11.6 Hz, 1H), 2.00 (s, 2H), 1.99 (s, 3H), 1.93 (s, 2H), 1.90 (s, 3H), 1.84 (d, *J* = 8.3 Hz, 1H), 1.76 (s, 4H), 1.73 (d, *J* = 4.9 Hz, 0H), 1.59 (d, *J* = 11.4 Hz, 1H), 1.42 (s, 1H), 1.31 (d, *J* = 7.4 Hz, 3H), 1.02 (dd, *J* = 10.0, 6.7 Hz, 3H), 0.97 – 0.73 (m, 20H).

Supplemental Scheme 7:

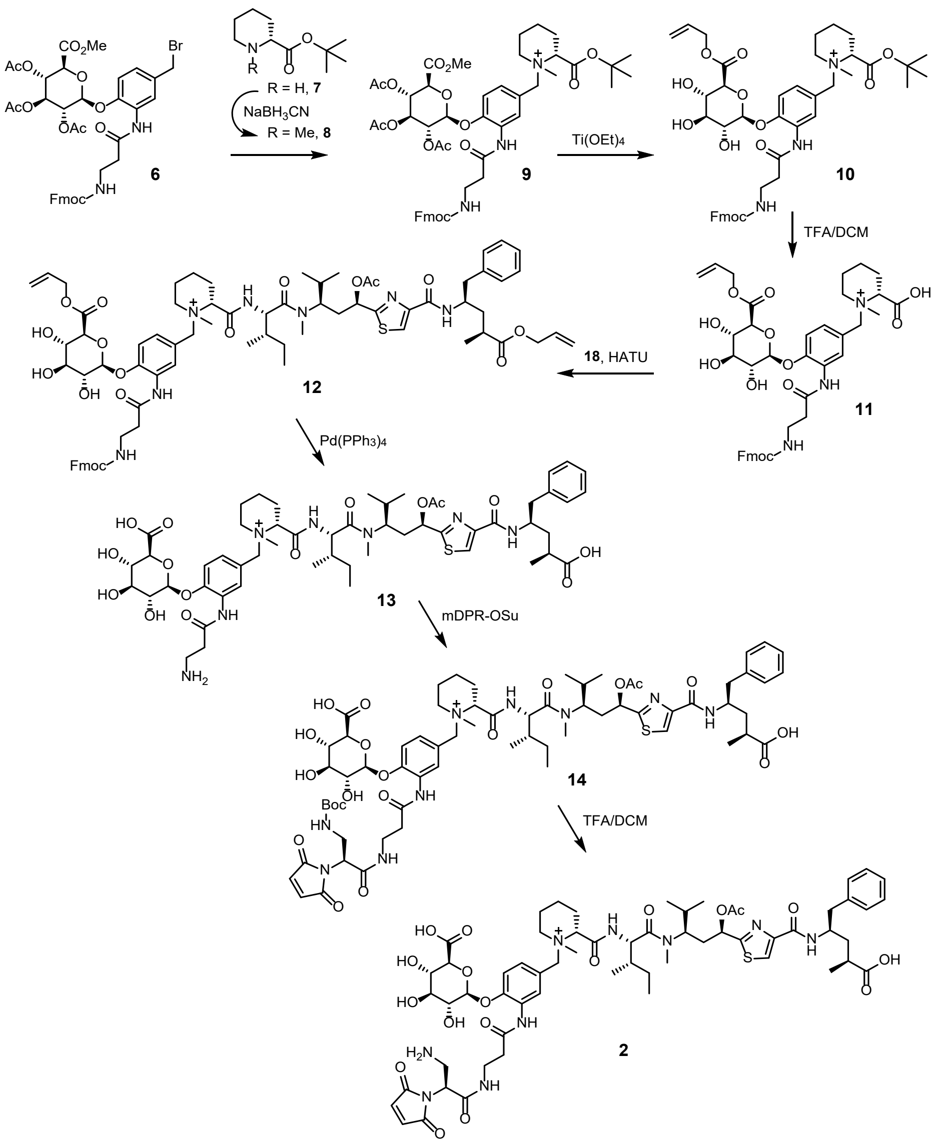

tert-butyl (R)-1-methylpiperidine-2-carboxylate (8): H-Pip-OtBu (**7**, 500 mg, 2.70 mmol) was taken up in MeOH (4.50 mL), AcOH (4.50 mL) and 37% CH_2O in H_2O (4.50 mL) and stirred for 20 minutes. $NaBH_3CN$ (509 mg, 8.10 mmol) added slowly as a solid to vigorous bubbling, stir for 30 minutes. The reaction was then poured into 200 mL saturated $NaHCO_3$ solution and extracted 3x with 200 mL DCM. The organic layers were washed with brine, dried over $NaSO_4$, and condensed under reduced pressure to provide **8** (516 mg, 96%) to be carried forward without further purification. Analytical UPLC-MS (system 2): t_r = 0.53 min, *m/z* (ES+) calculated 200.17 $[M+H]^+$; found 200.21. 1H NMR (400 MHz, Chloroform-d) δ 2.98 – 2.89 (m, 1H), 2.59 (dt, J = 10.0, 3.6 Hz, 1H), 2.26 (d, J = 2.6 Hz, 4H), 2.12 – 1.99 (m, 1H), 1.89 – 1.76 (m, 1H), 1.76 – 1.55 (m, 3H), 1.53 – 1.44 (m, 2H), 1.46 (s, 6H), 1.34 – 1.19 (m, 1H), 1.27 (s, 1H).

(2R)-1-(3-(3-((((9H-fluoren-9-yl)methoxy)carbonyl)amino)propanamido)-4-(((2S,3R,4S,5S,6S)-3,4,5-triacetoxy-6-(methoxycarbonyl)tetrahydro-2H-pyran-2-yl)oxy)benzyl)-2-(tert-butoxycarbonyl)-1-methylpiperidin-1-ium (9): A pressure vessel was charged with brominated glucuronide linker fragment **6** (104 mg, 128 μmol) and Mep-OtBu (**8**, 34 mg, 171 μmol) in anhydrous 2-butanone (1.71 mL). The

reaction vessel was flushed with N_2 and sealed. The reaction was then stirred and heated to 60 °C for 12 hours. The resulting mixture was cooled, condensed under reduced pressure, taken up in minimal DMSO and purified by preparative LC to provide **9** (97 mg, 82%). Analytical UPLC-MS (system 2): t_r = 1.32 min, *m/z* (ES+) calculated 930.40 $[M]^+$; found 930.49. 1H NMR (300 MHz, DMSO-d6) δ 8.96 (s, 1H), 8.03 (s, 1H), 7.94 – 7.85 (m, 2H), 7.69 (d, J = 7.4 Hz, 2H), 7.48 – 7.38 (m, 2H), 7.37 – 7.27 (m, 4H), 7.23 (d, J = 8.5 Hz, 1H), 5.74 (d, J = 7.9 Hz, 1H), 5.53 (t, J = 9.7 Hz, 1H), 5.29 – 5.17 (m, 1H), 5.08 (t, J = 9.7 Hz, 1H), 4.81 (d, J = 9.9 Hz, 1H), 4.76 – 4.52 (m, 2H), 4.31 (d, J = 7.6 Hz, 2H), 4.28 – 4.08 (m, 2H), 3.64 (s, 3H), 3.08 (s, 1H), 3.03 (s, 1H), 2.57 (s, 2H), 2.12 (s, 2H), 2.06 – 1.97 (m, 10H), 1.66 (s, 4H), 1.50 (d, J = 11.5 Hz, 10H), 1.11 (s, 3H).

(2R)-1-(3-(3-((((9H-fluoren-9-yl)methoxy)carbonyl)amino)propanamido)-4-(((2S,3R,4S,5S,6S)-6-((allyloxy)carbonyl)-3,4,5-trihydroxytetrahydro-2H-pyran-2-yl)oxy)benzyl)-2-(tert-butoxycarbonyl)-1-methylpiperidin-1-ium (10): A flame-dried flask was charged with Fmoc-GlucQ-Mep-OtBu (**9**, 97 mg, 104 µmol) in anhydrous allyl alcohol (2.09 mL) under N_2. $Ti(OC_2H_5)_4$ (87 µL, 417 µmol) was added and the reaction was heated to 80 °C with stirring for 2 hours. The reaction was then cooled to room temperature and poured into 50 mL 1M HCl. After resting for 45 minutes, the HCl was extracted 3x with 50 mL DCM. Resulting organics were washed with brine, dried over $NaSO_4$, condensed, and purified by preparative LC to provide **10** (42 mg, 48%). Analytical UPLC-MS (system 2): t_r = 1.18 min, *m/z* (ES+) calculated 830.39 $[M]^+$; found 830.49.

(2R)-1-(3-(3-((((9H-fluoren-9-yl)methoxy)carbonyl)amino)propanamido)-4-(((2S,3R,4S,5S,6S)-6-((allyloxy)carbonyl)-3,4,5-trihydroxytetrahydro-2H-pyran-2-yl)oxy)benzyl)-2-carboxy-1-methylpiperidin-1-ium (11): A flask containing Fmoc-Gluc(Allyl)Q-Mep-OtBu (**10**, 42 mg, 50 µmol) was cooled to 0 °C under N_2. A solution of 30% TFA in CH_2Cl_2 (2.5 mL) was added dropwise and stirred for 18 hours. The reaction was concentrated under reduced pressure, taken up in minimal DMSO and purified by preparative LC to provide **11** (25 mg, 64%). Analytical UPLC-MS (system 2): t_r = 1.05 min, *m/z* (ES+) calculated 774.32 $[M]^+$; found 774.42. 1H NMR (300 MHz, DMSO-d6) δ 9.15 (s, 1H), 8.27 (s, 1H), 7.89 (d, J = 7.4 Hz, 2H), 7.69 (d, J = 7.4 Hz, 2H), 7.47 – 7.26 (m, 4H), 7.19 (d, J = 7.0 Hz, 2H), 6.01 – 5.84 (m, 1H), 5.82 (s, 1H), 5.54 (s, 1H), 5.34 (dd, J = 17.4, 1.6 Hz, 1H), 5.21 (dd, J = 10.4, 1.6 Hz, 1H), 5.06 (d, J = 7.2 Hz, 1H), 4.73 – 4.60 (m, 4H), 4.30 (d, J = 6.8 Hz, 2H), 4.23 (d, J = 6.5 Hz, 1H), 4.17 (d, J = 9.5 Hz, 1H), 3.64 (s, 1H), 3.45 (s, 3H), 3.20 (s, 2H), 3.08 (s, 2H), 2.91 (s, 1H), 2.66 – 2.57 (m, 2H), 2.10 (s, 2H), 1.95 (s, 1H), 1.69 (s, 3H), 1.37 (s, 1H), 1.11 (s, 4H).

(2R)-1-(3-(3-((((9H-fluoren-9-yl)methoxy)carbonyl)amino)propanamido)-4-(((2S,3R,4S,5S,6S)-6-((allyloxy)carbonyl)-3,4,5-trihydroxytetrahydro-2H-pyran-2-yl)oxy)benzyl)-2-(((2S,3S)-1-(((1R,3R)-1-acetoxy-1-(4-(((2R,4S)-5-(allyloxy)-4-methyl-5-oxo-1-phenylpentan-2-yl)carbamoyl)thiazol-2-yl)-4-methylpentan-3-yl)(methyl)amino)-3-methyl-1-oxopentan-2-yl)carbamoyl)-1-methylpiperidin-1-ium (12): To a flask charged with H-Ile-Tuv(OAc)-Tup-OAllyl (**18**, 23 mg, 36 µmol) was added Fmoc-Gluc(Allyl)Q-Mep-OH (**11**, 28 mg, 36 µmol) and HATU (27 mg, 72 µmol) as solids followed by DMF (0.714 mL). N,N-Diisopropylethylamine (25 uL, 143 µmol) was added and the reaction was stirred at room temperature for 1 hour. The reaction was then taken up in DMSO and purified by preparative LC to provide **12** (23 mg, 46%). Analytical UPLC-MS (system 2): t_r = 1.39 min, *m/z* (ES+) calculated 1398.66 $[M]^+$; found 1398.81.

(2R)-2-(((2S,3S)-1-(((1R,3R)-1-acetoxy-1-(4-(((2R,4S)-4-carboxy-1-phenylpentan-2-yl)carbamoyl)thiazol-2-yl)-4-methylpentan-3-yl)(methyl)amino)-3-methyl-1-oxopentan-2-yl)carbamoyl)-1-(3-(3-minopropanamido)-4-(((2S,3R,4S,5S,6S)-6-carboxy-3,4,5-trihydroxytetrahydro2H-pyran-2-l)oxy)benzyl)-1-methylpiperidin-1-ium (13): Fmoc-Gluc(Allyl)Q-TubM-OAllyl (**12**, 21 mg, 15 µmol) was taken up in DCM (1.5 mL) stirring under N_2. $Pd(PPh_3)_4$ (3.5 mg, 3.1 µmol) and PPh_3 (1.6 mg, 6.1 µmol) were added as solids followed by pyrrolidine (20.1 µL, 245 µmol). The reaction was stirred for 2 hours at room temperature then taken up in 1 mL DMSO, condensed under reduced pressure, and purified by preparative LC to provide **13** (13 mg, 79%). Analytical UPLC-MS (system 2): t_r = 0.94 min, *m/z* (ES+) calculated 1096.53 $[M]^+$; found 1096.65.

(2R)-2-(((2S,3S)-1-(((1R,3R)-1-acetoxy-1-(4-(((2R,4S)-4-carboxy-1-phenylpentan-2-yl)carbamoyl)thiazol-2-yl)-4-methylpentan-3-yl)(methyl)amino)-3-methyl-1-oxopentan-2-yl)carbamoyl)-1-(3-(3-((S)-3-((tert-butoxycarbonyl)amino)-2-(2,5-dioxo-2,5-dihydro-1H-pyrrol-1-yl)propanamido)propanamido)-4-(((2S,3R,4S,5S,6S)-6-carboxy-3,4,5-trihydroxytetrahydro-2H-pyran-2-yl)oxy)benzyl)-1-methylpiperidin-1-ium (14): A flask was charged with H-GlucQ-TubM (**13**, 13.1 mg, 11.9 µmol) in anhydrous DMF (0.595 mL) to which mDPR(Boc)-OSu (4.6 mg, 11.9 µmol) was added under N_2. N,N-Diisopropylethylamine (8.3 uL, 47.8 µmol) was added and the reaction was stirred at room temperature for 3 hours. The reaction was then quenched with acetic acid (8.3 uL) and purified by preparative LC to provide **14** (5.2 mg, 33%). Analytical UPLC-MS (system 2): t_r = 1.20 min, *m/z* (ES+) calculated 1362.62 $[M]^+$; found 1362.75.

(2R)-2-(((2S,3S)-1-(((1R,3R)-1-acetoxy-1-(4-(((2R,4S)-4-carboxy-1-phenylpentan-2-yl)carbamoyl)thiazol-2-yl)-4-methylpentan-3-yl)(methyl)amino)-3-methyl-1-oxopentan-2-yl)carbamoyl)-1-(3-(3-((S)-3-amino-2-(2,5-dioxo-2,5-dihydro-1H-pyrrol-1-yl)propanamido)propanamido)-4-(((2S,3R,4S,5S,6S)-6-carboxy-3,4,5-trihydroxytetrahydro-2H-pyran-2-yl)oxy)benzyl)-1-methylpiperidin-1-ium (2): A flask charged with mDPR(Boc)-GlucQ-TubM (**14**, 5.2 mg, 3.8 µmol) was cooled to 0 °C under N_2. A solution of 10% TFA in CH_2Cl_2 (0.84 mL) was added dropwise and stirred for 4 hours. The reaction was then taken up in DMSO, condensed under reduced pressure, and purified by preparative LC to provide **2** (4.8 mg, 81%). Analytical UPLC-MS (system 2): t_r = 0.95 min, *m/z* (ES+) calculated 1262.56 $[M]^+$; found 1262.68. ^{1}H NMR (400 MHz, Acetonitrile-d_3) δ 8.40 – 8.35 (m, 2H), 7.88 (s, 3H), 7.23 – 7.19 (m, 2H), 7.15 (s, 3H), 7.05 (s, 1H), 6.57 – 6.49 (m, 3H), 5.55 – 5.51 (m, 1H), 4.84 (s, 0H), 4.70 (s, 1H), 4.23 (s, 1H), 3.99 – 3.95 (m, 5H), 3.80 (s, 2H), 3.69 (s, 1H), 3.60 (s, 2H), 3.17 (s, 1H), 3.16 (s, 16H), 2.99 (s, 7H), 2.90 (d, *J* = 1.4 Hz, 1H), 2.87 (s, 3H), 2.72 (d, *J* = 11.0 Hz, 1H), 2.24 (d, *J* = 1.5 Hz, 1H), 2.18 (s, 1H), 1.89 (d, *J* = 1.4 Hz, 5H), 1.76 – 1.71 (m, 2H), 1.59 (s, 1H), 1.47 (s, 1H), 1.16 (d, *J* = 11.5 Hz, 2H), 0.90 (d, *J* = 6.7 Hz, 5H), 0.81 (d, *J* = 7.4 Hz, 4H), 0.78 (s, 6H).

1. Jeffrey, S. C.; Andreyka, J. B.; Bernhardt, S. X.; Kissler, K. M.; Kline, T.; Lenox, J. S.; Moser, R. F.; Nguyen, M. T.; Okeley, N. M.; Stone, I. J.; Zhang, X.; Senter, P. D., Development and properties of beta-glucuronide linkers for monoclonal antibody-drug conjugates. *Bioconjug Chem* **2006,** *17* (3), 831-40.
2. Wipf, P.; Wang, Z., Total synthesis of N14-desacetoxytubulysin H. *Org Lett* **2007,** *9* (8), 1605-7.
